# New Approaches to Shifting the Migraine Treatment Paradigm

**DOI:** 10.3389/fpain.2022.873179

**Published:** 2022-09-06

**Authors:** Brian Johnson, Frederick G. Freitag

**Affiliations:** Department of Neurology, Medical College of Wisconsin, Milwaukee, WI, United States

**Keywords:** remote electrical neurostimulation, dihydroergotamine, CGRP, gepants, triptans

## Abstract

The standard of care paradigm for migraine treatment has been based almost exclusively on approaches that grew out of the happenstance use of market pharmaceuticals. Only methysergide, which has long since been removed from use for safety concerns, the ergotamine family of drugs, and the triptans were explicitly developed with migraine and other vascular headaches in mind. While the forward and innovative thinking to utilize the broad array of agents to treat migraine served millions well, their therapeutic efficacy was often low, and adverse event profiles were troublesome in the least. Advances in biochemical and molecular biology and the application of advanced “designing drugs” methods have brought about a potentially significant shift in treatment. The gepants have efficacies similar to the triptans but without vascular safety or medication overuse concerns. Preventative gepants offer innovative approaches to prevention and efficacy that exceed even the CGRP monoclonal antibodies. Those monoclonal antibodies brought rapid and highly effective outcomes across the spectrum of migraine. They outpaced older oral medication efficacy and eliminated most adverse events while potentially improving compliance with monthly or quarterly dosing. Other serotonin receptors beyond the 5HT1B and1D receptors have been targeted for decades. They now lead us to better formulations of dihydroergotamine for efficacy, convenience, and tolerability, and a 5HT1F-specific acute treatment like the gepants opens new options for acute management. Neuromodulation goes back to the mid-1800's. Our improved understanding of applied biomedical engineering has brought forward several tantalizing devices, including the application of currents distant from the target and patient regulated. Whether these advances change the paradigm of migraine treatment and standards of care remains to be seen, and issues such as cost and patient acceptance will help mold it.

## Introduction

Migraine headache has long been recognized as one of the most burdensome and prevalent diseases worldwide. It poses a significant stressor not only to patients but to healthcare systems. The estimated global prevalence of migraine is 11.6%. It varies slightly in different regions of the world, throughout different socioeconomic statuses, and across genders and races. Migraine headache ranks third among causes of years lived with disability worldwide (1–3). Treatments for headaches have advanced over the years. However, providing lasting and effective treatment for all headache types proves difficult for all practitioners. Only one-third of patients with chronic migraine (CM) are satisfied with their current therapy, which demonstrates this difficulty. The current standards anticipate that only 50% of patients receive at least a 50% improvement of their symptoms with any given pharmacologic regimen (a goal frequently used as a measurable endpoint in clinical trials) (4, 5). Recently, the US FDA approved various new classes of drugs for the treatment of episodic migraine (EM) and CM headaches. Importantly, these newer therapies aim not only to improve the efficacy of treatment but also to provide different delivery options and timeframes, and above all, to improve tolerability for patients. These newer therapies raise the potential for a shift in the paradigm of migraine treatment.

## Current Therapy For Migraine Headache

There are several published practice guidelines and guidance on behalf of the European Headache Federation and the Quality Standards Subcommittee of the American Academy of Neurology, The American Headache Society, among others (6–10). These guidelines help to provide a framework for the initial and ongoing treatment of migraine headaches.

## Abortive Therapy

The onset of migraine can vary within a group of patients or even in a single patient. Acute treatments primarily focus on providing relief of headache pain as well as treating other symptoms that can accompany migraines, such as photophobia, phonophobia, and nausea. Non-steroidal anti-inflammatory agents (NSAIDs) are considered the first-line therapy for acute treatment, including ibuprofen, naproxen, diclofenac, and ketorolac (11). The drawbacks of their use include cardiovascular, gastrointestinal, and renal effects. Patients commonly use the FDA-approved-for-migraine combination of acetaminophen, aspirin, and caffeine.

In patients who have failed NSAIDs or have disabling migraine, the triptans, such as sumatriptan, among others, target resolution of the acute migraine headache through their activity at the 5-HT1B/1D receptors. These agents are available *via* various routes of administration, including oral, intranasal powder, liquid nasal spray, and subcutaneous injection (12). Another alternative is dihydroergotamine (DHE). DHE is available as an intravenous, intramuscular, subcutaneous, or nasal spray.

## Preventive Therapy

Preventive therapy focuses on decreasing the number and frequency of attacks and improving the response to acute migraine medications. Medications must be taken on a schedule (usually daily), and dosages are adjusted to the response and tolerance of individuals. Divalproex sodium, sodium valproate, topiramate, metoprolol, propranolol, and timolol are considered the most effective prescriptions for migraine prevention (6). A special extract of Petasites (butterbur) is comparably effective for migraine prevention (7) without apparent risk of hepatotoxicity (13). This demonstrated efficacy and safety make it useful for patients who prefer a more natural remedy. Treatment of menstrual-related migraine with prevention uses cyclic dosing. Frovatriptan demonstrated effectiveness in the prevention of menstrual-associated migraine when taken for 6 consecutive days before period onset (14). Until now, onabotulinumtoxinA was the only FDA-approved treatment for CM. Treatment consists of a series of 31 injections given every 12 weeks. OnabotulinumtoxinA has been approved since 2010 and is well-tolerated and perhaps the most effective option (15).

## New and Emerging Therapies For Migraine

The standards of migraine therapy do have limitations. For some, it is adverse events or a lack of significant improvement. For others, it is medication overuse or inability to tolerate a delivery method or regimen. Advancements in headache therapies over the past decade have aimed not only to improve efficacy but also delivery and tolerability to ultimately improve patient satisfaction. These new approaches open options for clinicians treating patients with particularly refractory and difficult migraines. Emerging therapies include CGRP-receptor antagonists, CGRP antibodies, a 5-HT1F receptor agonist, and new deliveries for proven treatments in intranasal form and non-invasive neuromodulation.

## Cgrp-Receptor Antagonists (Small Molecule)

For years, researchers have known the role of calcitonin gene-related peptide (CGRP) in migraines (16). CGRP is a sensory neuropeptide that resides in the trigeminal ganglion, and its concentration in central venous blood has a direct correlation to migraine pain. From a pathophysiological approach, CGRP is a potent vasodilator. It plays an essential role in the sensory signaling pathway peripherally and centrally through the A-fiber sensory neurons of the trigeminal nerve that are involved in pain perception, and in satellite glial cells that modulate pain sensitivity and transmission when a pain signal is initiated (16). CGRP binds to four different receptor types: the CGRP receptor, the amylin receptors, the calcitonin receptor, and the adrenomedullin receptors (16). Receptor binding to CGRP occurs with particularly high affinity in two receptors—the CGRP receptor and the amylin receptor, AMY1 (17). Testing has revealed elevated levels of serum CGRP during migraine attacks, making the peptide and its high-affinity receptors particular targets for therapy in treating migraine headaches (18).

The trigeminal nerve fibers lie outside the blood–brain barrier. Researchers have theorized that circulating CGRP, acting outside the blood–brain barrier peripherally at CGRP receptors on perivascular trigeminal sensory fiber terminals or trigeminal ganglion, could magnify central sensory and nociceptive processing in the context of a trigeminal system that becomes sensitized by migraine headache attacks (19, 20). Hence, the CGRP-antagonist medications do not need to cross the blood–brain barrier, but rather act in the periphery to produce a response with small-molecule medication for acute and preventative treatment or with an antibody as a migraine preventive. They demonstrated safety in stable cardiovascular or cerebrovascular disease in clinical trials and, based on the information garnered from the mAbs, pose no risk of medication overuse.

Two small-molecule CGRP-receptor inhibitors, ubrogepant (21, 22) and rimegepant (23, 24) (colloquially referred to as the gepants), completed phase III studies and are now US FDA approved for the acute treatment of migraine with or without aura. Rimegepant is also approved for the prevention of migraine, as discussed further. Another inhibitor, atogepant, approved in 2021 by the US FDA is a daily oral medication used for EM prevention (25, 26).

Ubrogepant was studied in the ACHIEVE I and II trials, with the co-primary endpoints of pain freedom and absence of the participant-designated most bothersome migraine-associated symptom (MBS) chosen among photophobia, phonophobia, and nausea. Both primary outcome measures were met. As compared to placebo, patients taking 25, 50, or 100 mg doses reported statistically significant pain freedom after 2 h, and only patients assigned 50 and 100 mg doses reported a significant improvement in their MBS (27). A *post-hoc* analysis provided important data regarding other measures of improvement that were patient-reported, including scores on the Functional Disability Scale (FDS), overall satisfaction with study medication, and patient global impression of change (PGIC). Of these other measures, the patient-reported FDS was noteworthy with a statistically significant proportion of patients reporting no disability (i.e., able to function normally) in the ubrogepant 50 and 100 mg groups as compared to the placebo group at the 2, 4, and 8-h marks. The most commonly reported side effects were nausea and dizziness. Of particular concern in the development of the CGRP-receptor antagonists has been the development of hepatic injury. This concern came from findings with first-generation gepants (28, 29), which showed a possible correlation with elevated ALT/AST up to 3 × the upper limit of normal. Both ubrogepant and rimegepant have completed phase III trials showing efficacy and safety with no signs of hepatic injury (30).

Rimegepant is used for the acute treatment of migraine with or without aura. The phase III study used the same outcome measures as the ubrogepant studies, with co-primary endpoints of freedom of pain at 2 h and the absence of the MBS. The study found statistically significant improvement in both these metrics (24). There were 21 secondary endpoints in three broad categories included in the trial data. First, general relief of migraine symptoms that focuses on freedom from photophobia, phonophobia, and nausea, along with freedom from pain. Second, endpoints that reflect early action, with freedom from pain and the MBS at 60- and 90-min timepoints. Third, the endpoints that focus on the durability of the effectiveness of the drug for pain relief, freedom from pain, and the MBS from 2 to 24 h and 2 to 48 h. Rimegepant was superior to placebo in all the secondary endpoint measurements mentioned above, except for freedom from nausea and pain relapse from 2 to 48 h.

Several meta-analyses have been conducted on these new gepants (31, 32) of rimegepant and ubrogepant. One meta-analysis compared both of them and lasmitidan (33). These consistently demonstrate the superiority of the active agents over the placebo. The analysis showed that rimegepant is somewhat more effective than ubrogepant and has similar tolerability. Lasmitidan appears to be more effective than either of these for relieving migraine and other bothersome symptoms, but the rate of adverse events is substantially greater (Table 1).

**Table 1:** Efficacy and tolerability of acute treatment medications.

**Drug**	**Dose**	**2H pain free (%)**	**2H freedom of most bothersome symptom (%)**	**2–24 H sustained pain freedom (%)**	**Adverse events**
Ubrogepant	50 mg	19.2–21.8	38.6–38.9	12.7–14.2	Nausea 2% Somnolence 2%
Ubrogepant	100 mg	21.2	37.7	15.4	Nausea 4% Somnolence 3% Dry mouth 2%
Ubrogepant	Placebo	11.8–14.3	9.9–11.5	8.2–8.6	Nausea 2% Somnolence 1% Dry Mouth 1%
Rimegepant ODT	75 mg	21.2	35.1	13.5 (2–48 h)	Nausea 2% Hypersensitivity 1 <
Rimegepant ODT	Placebo	10.9	26.8	5.4 (2–48 h)	Nausea 0.4
Lasmiditan	50 mg	28.3	40.8	ND	Dizziness, nausea. and/or vomiting 3% Muscle weakness 1%
Lasmiditan	100 mg	28.3–31.4	41.2–44	ND	Dizziness 15% Fatigue 4% Paresthesia 3% Sedation 6% Nausea and/or vomiting 3% Muscle weakness 1%
Lasmiditan	200 mg	31.8–38.8	40.7–48.7	ND	Dizziness 17% Fatigue 6% Paresthesia 9% Sedation 7% Nausea and/or vomiting 4% Muscle weakness 2%
Lasmiditan	Placebo	15.3–21	29.6–33.2	ND	Dizziness 3% Fatigue 1% Paresthesia 2% Sedation 2% Nausea and/or vomiting 2% Muscle weakness 0%
Sumatriptan nasal spray with permeation enhancer	10 mg	43.8	70.7%	ND	9.7% any AE dysgeusia 8.1%
Sumatriptan nasal spray with permeation enhancer	Placebo	22.5	39.5%	13.8	ND
Liquid nasal dihydroergotamine with Precision Olfactory Delivery		35–39	51–59	35	16.7% nasal congestion
Liquid nasal dihydroergotamine with Precision Olfactory Delivery	Placebo	ND	ND	ND	ND
Dry powder nasal dihydroergotamine	3.9 mg	20	39.5	ND	ND
Dry powder nasal dihydroergotamine	5.4 mg	19.5	40	ND	ND
Dry powder nasal dihydroergotamine	Placebo	16	33	ND	ND
Celecoxib oral solution	120 mg	32.8–41.2	58.1–63.4	40.2	Overall, 4.5–13.3 Nausea 0.8–3.2 Dysgeusia 0.8–4.2
Celecoxib oral solution	Placebo	22.1–31.1	43.9–50.0	27.2	Overall, 5.6–8.9 Nausea 1.2–1.8 Dysgeusia 0.8–1.4

*ND, No Published Data*.

Rimegepant is also approved for preventive use (34). This dual-use of rimegepant for acute and preventative treatment of migraine opens new and innovative approaches for clinicians and patients. One set of these approaches is to use it as either only an acute treatment or as a preventative treatment for migraine. It can be used as a typical preventative, because of its long pharmacological half-life (23), and can be administered every other day. However, another approach that lends itself to this dual-use treatment is a “pre-emptive preventative.” It means that patients who have identified events that trigger their migraine attacks could take a dose the day prior and therefore avert the migraine. Common triggers such as weather change, stressful life events, and letdown periods may be treated with a single dose, thereby, avoiding a significant risk of an attack occurring. In menstrually related migraine, a pre-emptive approach, even if it requires additional days of treatment based on the length of the woman's susceptibility to multiple days of menstrual-related migraines, can be successful. One of the authors demonstrated with another long half-life agent, dihydroergotamine, where many subjects required only the initial day of treatment and had no further migraine attacks associated with that menstrual cycle (35). In these pre-emptive preventative treatment scenarios, the patient would still be able to take another dose of rimegepant if a migraine occurred or use another migraine-specific agent, such as a triptan or ergot derivative.

It is also being studied as a treatment for refractory trigeminal neuralgia (36). A recently published case report presents striking evidence of two patients enrolled in a clinical trial of rimegepant, who went on to use erenumab (a monoclonal antibody described below) and then gained significant improvement in their EM as compared to their baseline (37). A study performing a comparative analysis of trials of rimegepant for prevention compared to galcanezumab and erenumab (38) demonstrated comparable efficacy and tolerability of rimegepant vs. both mAbs. In a recent case series (39), there was no evidence of adverse interaction between gepants used acutely and mAb for migraine prevention. Additionally, we did not see any enhancement of effects of the mAb on improved response with a gepant nor that the use of a gepant on an as-needed basis improved the preventative response of the mAb. Importantly, using a gepant in patients on erenumab, with both acting on the same CGRP receptor, resulted in highly successful acute relief of breakthrough migraine attacks in patients incompletely remitted on erenumab. Though not confirmatory of any trend, this type of evidence provides useful data that can help guide the ideas and methods of future investigations into using these medications for migraine treatment.

Atogepant is another CGRP-receptor antagonist taken orally; however, the primary goal is to use it as a preventative for EM (25). The primary trial endpoint was a reduction of mean monthly migraine or probable migraine headache days. Atogepant showed statistically significant improvement at 10 mg QD, 30 mg QD, 60 mg QD, and 30 mg and 60 mg BID dosing vs. placebo (26). Compared to other oral CGRP-blockers, this new daily oral preventive therapy could significantly improve the headache profiles of many patients unwilling to take, or unable to tolerate, other routes of administration.

## Cgrp-Receptor and Peptide Antibodies

As previously discussed, CGRP as a neuropeptide plays a major role in migraine pathophysiology. Much like the small-molecule medications designed to block receptor binding, there are monoclonal antibodies (mAbs) with action on the same pathway. They either block the CGRP-receptor specifically or bind to the neuropeptide itself. Unlike the small-molecule medications, which come in tablet or capsule forms, the mAbs are peptides with long half-lives delivered parenterally (40). Improved patient compliance is likely by dosing these medications once monthly (or IV infusion every 3 months for eptinezumab), as compared to standard oral preventive therapies dosed daily. Four mAbs have been approved by the US FDA for migraine prevention. Galcanezumab, fremanezumab, and eptinezumab are humanized mAbs that selectively bind directly to CGRP to disable the neuropeptide as an active ligand, whereas erenumab is a fully human mAb directed toward the CGRP receptor (41, 42). Other than eptinezumab, these drugs use an autoinjector device for delivery. Fremenzumab, like eptinezumab, allows for injections every 3 months.

These four mAbs have been US FDA approved for the prevention of both episodic and chronic migraine. They share similarities but also differences in their origin and structure (42). Eptinezumab, fremanezumab, and galcanezumab are humanized mAbs, while erenumab is a human antibody. The three humanized antibodies target CGRP itself in the circulation, blocking the ability of the neuropeptide ligand from accessing the receptor. However, erenumab binds directly to the trigeminal CGRP receptor to disable the neuropeptide from binding to the receptor.

The mAbs offer the benefit from a pharmacokinetic perspective in that they have long half-lives approximating a month and that they do not undergo hepatic degradation, the issue that arose with the early gepants. This combination avoids the issue of daily dosing seen with most oral preventatives, thereby at least in theory improving patient compliance while at the same time avoiding potential metabolic issues related to drug–drug interactions in hepatic metabolism. However, they do lack the convenience and preferred delivery route of oral administration.

The mAbs belong to one of three IgG classes (43). Mass production utilizes hybridomas from different sources made up of cells in continuous division. This allows for a clone responsible for producing a single type of antibody. Amino acid sequencing distinguishes the different regions and origins of the antibody. With this technique, chimeric rodent fragment antigen-binding (Fab) region, human Fragment crystallizable (Fc) region, humanized mouse complementarity determining regions (CDR), and human Fab are identified and differentiated from a human antibody, where the whole sequence of the human antibody gene is produced in the mouse. The CDRs determine the ability of the antibody to attach to the ligand (CGRP neuropeptide) or the CGRP trigeminally located receptor. Only erenumab is human, while the CDRs are from the mouse for fremanezumab and galcanezumab and rabbit for eptinezumab. Erenumab is a humanized IgG2λ, eptinezumab is a humanized IgG1k, fremanezumab is a humanized IgG2k, and galcanezumab is a humanized IgG4. All three subcutaneously administered antibodies have great variability in their pharmacokinetic parameter of *T*_max_. Erenumab has a range of *T*_max_ from 3 to 14 days, fremanezumab's range is from 3 to 20 days, and galcanezumab's range is between 7 and 14 days. Eptinezumab reaches its *T*_max_ in 4.8 h and is given intravenously. Compared to the other three mAbs, it has a faster association with the CGRP ligand yet dissociates slower than the other two ligand-specific mAbs. None of the mAbs readily cross the blood– brain barrier (BBB). CGRP receptors are found outside the BBB. Edvinsson et al. (44) recently examined the relationship between those neurons in the trigeminal ganglion expressing CGRP in unmyelinated sensory C-fibers and CGRP receptors found on myelinated Aδ sensory fibers, as it had been theorized that anti-migraine drugs might act to prevent CGRP binding to trigeminal Aδ-fibers in the periphery. They felt this could cause alterations in the regulation of excitatory potentials in neuronal cell bodies and modulation of conduction potentials in sensory fibers. Aδ-sensory nerves contain the nodes of Ranvier along with other nodal-associated regions. They have differences in the compositions of ion channels and roles played in neuronal conduction. Contactin-associated protein 1 (CASPR) is found only in the paranodal region of the neurons to allow the study of this specific region. Their study demonstrated that axon–axon CGRP signaling occurs between C-fibers and the Aδ-fibers in the trigeminal ganglion and the dura mater. They also showed that CGRP receptors co-localize with CASPR in the para-Ranvier nodes, and the pattern of nodes was in close proximity to CGRP-positive boutons on C-fibers. It means that CGRP regulates transmission in trigeminal nerves *via* axon–axon signaling and is a novel site of action for treatment. Erenumab before binding must enter the vascular endothelial cells through pinocytosis. It targets a fusion protein of the extracellular of the human G protein-coupled receptor calcitonin receptor-like receptor (CALCRL), which is required for the receptors for CGRP and adrenomedullin, along with RAMP1, which includes the CGRP binding pocket (43). It is highly specific for the CGRP receptor with a 5,000-fold greater selectivity for the CGRP receptor without evidence of producing either an agonist nor antagonist effect on other human calcitonin family receptors such as adrenomedullin, calcitonin, and amylin. Fremanezumab may have a differential difference between different blood vessels in examining CGRP-induced vasodilation (45). CGRP activity is not relegated only to vascular actions but may act directly on neurons, glial cells, and the glymphatic system (46).

Each of the CGRP mAbs has been the subject of multiple clinical trials to determine the safety and efficacy of these agents. The phase 3 trials have been used for the registration of these agents in various countries. Each of these trials has been subject to additional papers to look at primary outcomes and safety measures in addition to examination of subsets of these groups and long-term safety and in some cases efficacy studies leading to a truly dizzying array of publications and data. Because of the nature of the registration trials in the US, none of these agents were compared with treatments that might be considered standard of care for migraine prevention (Table 2).

**Table 2:** Comparative efficacy and tolerability of CGRP mAb's with topiramate and onabotulinumtoxinA.

	**Epti EM**	**Epti CM**	**Eren EM**	**Eren CM**	**Frem EM**	**Frem CM**	**Gal EM**	**Gal CM**	**TPM 50–200 mg EM**	**OnBTA 16–190.5 u EM**	**OnBTA 100–190 u CM**
50% responder rate odds ratio	1.29 (0.67–2.49) to 2.16 (1.47–3.39	1.80 (1.07–3.03) to 2.09 (1.71–2.38)	1.12 (0.65–1.94) to 5.12 (2.42–10.81)	1.57 (1.12–2.23) to 2.27 (1.52–3.39	2.06 (1.46–2.92) to 3.21 (1.77–5.81)	2.44 (1.32–4.54) to 3.11 (2.23–4.35)	1.55 (0.84–2.83) to 3.38 (1.95–5.95)	2.10 (1.47–2.99) to 4.58 (2.02–10.32)	1.27 (0.70–2.31) to 32.5 (2.79–378.66)	0.78 (0.52–1.18) to 2.56 (0.99–6.63)	1.28 (0.98–1.67) to 2.57 (0.52–12.76)
Overall 50% responder odds ratio	2.33 (2.08–2.60)								2.67 (1.94–3.68)		1.28 (0.98–1.67)
AE %	57.7–59.7	47.8–56.1	48.4–66.8	45.5	50.0–62.4	50.5–70.5	51.0–68.3	51.0–57.5	44.5		57.3
Drop out %	3.5–4.3	1.0–2.0	1.1–3.6	3.5	1.0–9.1	1.0–1.7	2.4–17.8	0.9–2.4	29.0		3.4

*Epti, Eptinezumab; Eren, Erenumab; Frem, Fremanezumab; Gal, Galcanezumab; TPM, Topiramate; OnBTA, OnabotulinumtoxinA; EM, Episodic Migraine; CM, Chronic Migaine; AE, Adverse events*.

Galcanezumab gained US FDA approval in 2018 with the indication of preventive treatment of migraine. The EVOLVE I and II trials (47, 48) were phase III trials that proved their efficacy and safety over 6 months, with a 5-month follow-up after the last injection. The primary endpoint of these studies was the overall mean change in monthly migraine headache days (MHDs). EVOLVE I showed a significant reduction of mean monthly MHDs by 4.7 days (120 mg), but the 240 mg dose produced a reduction of 4.6 days, which was not significant compared to 2.8 days with placebo (47). Likewise, in the EVOLVE II trial, the dose of 120 mg was statistically superior, but the dose of 240 mg did not reduce MHD as compared to the placebo (48). Key secondary endpoints were 50, 75, and 100% response rates; monthly MHDs with acute migraine medication use; Patient Global Impression of Severity rating; and the Role Function-Restrictive score of the Migraine-Specific Quality of Life Questionnaire, all of which showed superiority vs. placebo for the 120 mg dose. *Post-hoc* evaluation of this data showed that galcanezumab was effective in patients with both high-frequency EM (defined as 4–7 monthly MHDs) and low-frequency EM (defined as 8–14 monthly MHDs) (49). Another placebo-controlled phase III trial, REGAIN, presented the same primary endpoint goal in patients with CM. Medication administration used 120 or 240 mg doses over 3 months with a placebo control and a 9-month open-label extension (50). The 120 mg dose of galcanezumab was statistically superior to the placebo in this study of CM prevention.

Fremanezumab is another approved humanized mAb against CGRP, which has undergone phase III trials for the preventative treatment of EM and CM. In a 12-week trial, fremanezumab met the primary endpoint of mean change from baseline in the average monthly MHDs (defined in this study as days in which headache pain lasted ≥4 consecutive hours typically requiring medication) as well as two times as many patients reporting 50% reduction in the average number of headache days per month when compared to placebo (51). The FOCUS trial reported that fremanezumab, whether dosed monthly (225 mg) or quarterly (675 mg), showed a significant reduction of baseline monthly MHDs in patients with EM and CM who had previously failed two to four other classes of migraine preventive medication (45).

Eptinezumab is another humanized mAb against CGRP, tested in the PROMISE 1 and PROMISE 2 studies (52, 53), which aimed to prove efficacy in patients with EM or CM, respectively. In contrast to the other mAbs mentioned here, eptinezumab dosing is a 100-mg IV formulation every 3 months. A primary endpoint of change in the mean number of monthly MHDs occurred in both studies. An indication for use every 3 months, as opposed to a monthly regimen required with the other mAbs, may benefit some patients who struggle with the tolerability or compliance that monthly injections require.

Erenumab is the only mAb that targets the CGRP receptor instead of the neuropeptide itself (41). The ARISE and STRIVE trials tested erenumab in the treatment of EM with the primary endpoints of change in monthly MHDs and change from baseline to 4 to 6 months in the mean number of monthly MHDs, respectively (54). ARISE demonstrated significant efficacy and safety vs. placebo at doses of 70 mg injected monthly. STRIVE showed efficacy and safety with 70 and 140 mg monthly doses and similar secondary endpoints, including a significant reduction of 50% of monthly MHDs (55). Erenumab has also been proven effective and tolerable in CM (56) and in patients with CM who have previously failed preventive therapy (57).

There are no head-to-head trials of the mAbs either among themselves or compared to other standard care medications (Table 2). Therefore, the use of meta-analysis may be the best comparator approach to utilize. While there have been multiple meta-analyses for comparing the mAbs, only two compared the three subcutaneous administered versions, which were available when the studies were conducted (58, 59). Two others examined all four mAbs. One was an analysis of the four mAbs vs. topiramate in episodic migraine (60). One reported both individual mAb reports vs. multiple onabotulinumtoxinA trials but also combined the outcomes of the mAb trials vs. the onabotulinumtoxinA trials (61). One other examined the three subcutaneous mAbs vs. topiramate and onabotulinumtoxinA in episodic and chronic migraine in a number needed to treat for a 50% responder rate (NNT50%) and likelihood to help or harm 50% rate (LHH50%) (62).

The two meta-analyses of the subcutaneous mAbs, shown in the analysis by Shi et al. (59), from 11 randomized controlled trials (RCTs) of over 6,000 patients administered with all three subcutaneous mAbs, including alternative dosing for galcanezumab and fremanezumab were statistically superior to placebo and the erenumab 70 mg dose in reducing monthly migraine days (MMD). Alasad and Asha (58) examined the trial of erenumab 70 mg and monthly dosing of fremanezumab, and the 120 mg dose of galcanezumab in 13 RCTs of nearly 7,000 patients. Combined they produced significantly greater reductions in MMD at the 3 monthly timepoints compared to placebo. These results were similar across each of the three mAbs individually as well vs. placebo. They found no uptick in treatment-related adverse effects (TREA) compared to placebo.

Deng et al. (63) analyzed all four of the mAbs in episodic migraine involving 4,400 patients from 11 phase II and III trials. All four produced statistically significant results compared to placebo, individually and combined for a reduction in MMD. Eptinezumab had the highest proportion of 50% reduction in MMD and erenumab the lowest. TREAs were relatively fewest for erenumab and highest for galcanezumab, but none were statistically significant. Wang et al. (18) reviewed 118 RCTs and over 8,900 patients. Among all the treatments, fremanezumab had the highest probability of being ranked first in reduced MMD, followed by galcanezumab, erenumab, and eptinezumab. However, between-drug comparisons did not show significant differences. Galcanezumab was more likely to cause at least one more TREAs than placebo and had the highest probability of being ranked first in TEAEs, followed by fremanezumab, eptinezumab, and erenumab. The relative ratio of 50% rates in reduced MMD was highest for fremanezumab and lowest for eptinezumab. The relative rate of serious adverse events was highest for galcanezumab and lowest for erenumab compared to placebo.

The four mAbs were compared to topiramate in episodic migraine in a meta-analysis by Overeem et al. (60). They included 13 trials of almost 7,600 patients. There were three trials with erenumab, two with galcanezumab, two with fremanezumab, one with eptinezumab, and five with topiramate. They failed to find a statistically significant benefit for the mAbs when compared to topiramate for efficacy. However, the incidences of several common topiramate-related side effects were significantly more common when compared to the mAbs. The 50% rates for NNT were 6 for the mAbs and 7 for topiramate, for numbers needed to harm (NNH), 130 for the mAbs and 9 for topiramate, and for the LHH 24.3 to 1 for the mAbs but 1.8 to 1 for topiramate.

The comparison of the mAbs to onabotulinumtoxinA involved 10 studies pooled with a total of 6,325 patients in the meta-analysis by Lu et al. (61). The pooled analysis of reduction in MMD included 4,522 subjects in mAb trials and 1,384 in the onabotulinumtoxinA trials. The placebo-subtracted mean difference was −2.13 for the mAbs and −1.95 for onabotulinumtoxinA, which was not statistically significant. Along the same lines, the change in monthly headache days was −1.94 for the CGRP mAbs compared to −1.86 for onabotulinumtoxinA. The 50% responder rate used a relative rate comparison of 3,401 patients in mAb trials vs. 621 in onabotulinumtoxinA trials. Again, there was a non-statistically significant difference in favor of the CGRP mAbs over onabotulinumtoxinA, of −1.56 vs. −1.31. Adverse events also trended in favor of the mAbs over onabotulinumtoxinA.

A more complex analysis of the three subcutaneous CGRP mAbs vs. both onabotulinumtoxinA and topiramate in both the episodic and chronic migraine populations was recently published by Frank et al. (62). They included 32 trials of 13,302 patients in mAb trials and 1,989 from onabotulinumtoxin trials. They examined the 50% responder rate and calculated the odds ratios for the various agents. The four CGRP monoclonal antibodies showed odds ratios from 1.12 (0.65–1.94) to 5.12 (2.42–10.87).

## 5-Ht1F-Receptor Agonists

The 5-HT1 (serotonin) receptors in the CNS have been a focus of study for decades. The first specific antimigraine drugs developed were the ergot alkaloids, which demonstrate non-specific agonism for 5-HT1 receptors (63) with a central effect through activation of 5-HT1B, 5-HT1D, and 5-HT1F receptors on trigeminal nerve terminals, inhibiting the release of vasoactive peptides preventing vasodilatation in migraine (64). Triptans, as a class, function as agonists peripherally at 5-HT1B receptors, which mediate a weak vasoconstrictive effect, and centrally at 5-HT1D receptors in the trigeminal nuclei of the brainstem to decrease the transmission of pain signals (64). Both the ergots and the triptans' main therapeutic action is through 5-HT1D- mediated neurotransmitter reduction, but their effects are limited by their action on vasoconstriction of cranial blood vessels through the activation of 5-HT1B receptors (65). The use of these 5-HT1B receptor agonist medications is contraindicated in patients with certain cardiovascular diagnoses because of their vasoconstrictive effect (66). The development of a safe and effective 5-HT1F agonist migraine therapy has been long-awaited to provide an option to this subset of patients, as *in-vitro* studies have shown that 5-HT1F does not mediate a vasoconstrictive effect on cerebral or coronary arteries (67).

Lasmiditan is a high-affinity, highly selective 5-HT1F receptor agonist that acts on the trigeminal system without causing vasoconstriction because of its low affinity for 5-HT1B receptors (68). It is approved for the acute treatment of migraine with and without aura. In two-phase III placebo-controlled trials, SAMURAI and SPARTAN (69, 70), patients took the study medication within 4 h of the onset of a migraine, with the primary endpoints being headache pain freedom and freedom from the MBS after 2 h. These trials showed efficacy and safety in both these points for all three studied doses of 50, 100, and 200 mg. The *post-hoc* analysis demonstrated a sustained response at 24 and 48 h (71). A long-term trial, GLADIATOR, allowed entry of patients from the SAMURAI and SPARTAN trials. This study measured change in the Migraine Disability Assessment Scale (MIDAS) over 12 months (72). The mean baseline MIDAS score was 29. The scores significantly decreased at 3, 6, 9, and 12 months by 12.5 in the 100 mg group, and by 12.2 in the 200 mg group. Importantly, there were no meaningful reports of cardiovascular events across all groups. A *post-hoc* pooled analysis (73) of the SAMURAI and SPARTAN trials revealed that, of 4,439 patients who received ≥1 dose of the study drug, a total of 3,500 patients (78.8%) had ≥1 cardiovascular risk factor (CVRF), and 1,833 patients (41.3%) had ≥2 CVRFs at baseline. No statistical difference was found in the frequency of likely CV treatment-emergent adverse effects (TEAEs) with or without CVRFs. The only likely CV TEAE seen across patients with ≥1, ≥ 2, ≥ 3, or ≥ 4 CVRFs was palpitations (74). However, a link between lasmiditan and driving impairment occurred around 1.5 h post-dose. This finding resulted in a black box warning that patients should not drive for at least 8 h after use (74). It was included in the meta-analysis reported above (Table 2).

## Neuromodulation

Invasive and non-invasive neuromodulation therapy has offered a non-pharmacologic option for patients. Deep brain stimulation (DBS), sphenopalatine ganglion stimulation (SPGS), vagal nerve stimulation (VNS), transcranial magnetic stimulation (TMS), occipital nerve stimulation (ONS), and supraorbital nerve stimulation (SNS) have all shown promising results, with some options more viable than others (75).

One newer therapy is the Nerivio^TM^ armband, a non-invasive remote electrical neuromodulation (REN) device. This wearable device is applied for 45 min to the upper lateral arm between the bellies of the lateral deltoid and the triceps and provides stimulation to peripheral nerves to induce a conditioned pain modulation response (76). The stimulation directed at C and Aδ fibers sensory fibers produces a level of electrical stimulation that remains below the perceptual pain threshold, providing a pain-free experience for the patient (77). In randomized placebo-controlled trials, researchers studied subjects with 2–8 migraines per month. The subjects applied the device for 30–45 min, beginning within 1 h of attack onset, which showed that 66.7% compared to 38.8% of sham had pain relief. The primary endpoint was the proportion of individuals achieving pain relief at 2-h post-treatment, with secondary endpoints of relief of the MBS and pain freedom after 2 h, which showed 37.4% with REN and 18.4% with sham. A significant number of subjects met the primary endpoint and the MBS endpoint by a two-to-one margin vs. a placebo (sham) device (78). From a clinician's perspective, the growing field of non-invasive, non-pharmaceutical options is an exciting prospect.

A second device that combines occipital and trigeminal external stimulation (Reviolon) (79) showed in a controlled trial that complete migraine occurred with 46% of active treatment compared to 11.8% with the sham device.

## New Delivery Modalities

One significant barrier to the effective treatment of individuals with migraine is the tolerability of medication or therapy. Nausea is a significant symptom that frequently accompanies migraine headaches and may prohibit the use of oral medications in some. Advancing therapies also aim to improve delivery and tolerability by adjusting the administration route of established treatments, such as the triptans.

## Sumatriptan Nasal Spray

TosymraTM (DFN-02) is a nasal spray formulation of 10 mg sumatriptan combined with a permeation enhancer (0.20% 1-O-n-dodecyl-b-D-maltopyranoside) for acute migraine treatment aimed at improving the absorption, thus augmenting the pain-free response (80). Trials compared this new compound to the previously available intranasal liquid and injectable forms of sumatriptan and a significant improvement was achieved in the absorption based on improved time of response (81). Outcomes of placebo-controlled trials found 2-h pain free to be 43.8% active vs. 20.5% for placebo (*P* = 0.025) (82). By comparison the Cochrane Review (83) reported for the 20-mg original nasal spray 32% (283/891; range 25–57%) 2-h pain-free for the active vs. placebo 11% (52/488; range 4–25%).

## Dihydroergotamine Nasal Preparations

DHE is a synthetic migraine treatment used for years as a potent IV formulation to abort refractory migraines in a hospital or infusion-center setting. It has a unique pharmacologic profile of interaction with a broad array of loci believed to be important in migraine. A recent study (84) demonstrated that DHE (10 μM) exhibited agonist activity at the following receptors: Adrenoceptor α2B, CXCR7, Dopamine D2, D5, 5HT1A/1B/2A/2C/5A, binding with high affinity to the 5HT1B, Adrenoreceptor α2B, and Dopamine D2receptors. DHE also exhibited antagonist activity at the following receptors: Adrenoceptor a1B, a2A, a2C, CALCR-RAMP2, Dopamine D1, D3, D4, D5, and 5HT1F. Further work showed that DHE did not bind to 5HT3 and 4E receptors at concentrations up to 300 nM. Two newer formulations and delivery devices have been developed for the intranasal administration of DHE. One is STS-101, a dry powder spray, and the other is Trudhesa™, a liquid with an altered anatomic approach to delivery. STS-101 failed to achieve the 2-h endpoints despite having an excellent pharmacokinetic profile with faster absorption and peak plasma concentrations as compared to other DHE nasal delivery options. Trudhesa™ delivers DHE to the upper nasal space using a Precision Olfactory Delivery (POD^®^) device, where it is rapidly absorbed with ~1/10th *C*_max_ of IV DHE yet displays IV-like plasma levels from 20 min onward (85). The efficacy and safety were examined in a long-term study of up to 52 weeks. A total of 37% of migraine attacks were reported pain-free at 2 h, with 35 and 32% sustained pain freedom at 24 and 48 h (86) nasal adverse events occurred in 162 patients (45.8%) reported a nasal-related TEAE in the 24-week FSS, of which none were serious. The most common (incidence 3.10%) nasal-related TEAEs were nasal congestion (*n* = 59, 16.7%) and upper respiratory tract infection (URTI; *n* = 38, 10.7%) (87). Another formulation of DHE utilizes a dry powder for administration, STS 101. It demonstrated excellent pharmacokinetics (88, 89). STS101 achieved rapid and sustained high drug exposure with low variability. STS101 had a higher *C*_max_ than intranasal liquid sprays Migranal™ and Trudhesa™ and approached that of the orally inhaled MAP0004. The STS101 AUC0-2hr was 2-fold or greater than for Migranal™, Trudhesa™, and MAP0004. The STS101 AUC0-inf was 2-fold or greater than for Migranal™, Trudhesa™, and MAP0004. STS101 achieved 83% of the total drug exposure (AUC0-inf) of IM DHE and was comparable to IV DHE. STS101 achieved higher cumulative drug exposure than Migranal™, Trudhesa™, and MAP0004 by ~30 min and all timepoints thereafter. Plasma concentrations and AUC values for Migranal™ were similar across multiple historical studies and in the STS101 phase 1 study, but were lower in the Trudhesa™ phase 1 study. Their pivotal trial failed to meet the FDA 2-h primary endpoint but did at 3 h. Several factors may have influenced these, including subject selection, COVID issues, and device delivery issues (unpublished results). It is undergoing a new pivotal trial attempting to correct the deficiencies.

## Celecoxib

Nonsteroidal anti-inflammatory drugs (NSAIDs) have been commonly used for the treatment of migraine. Their mechanism of action and effects on migraine may be related to their effects on inflammation. This link is related to prostaglandins in the smooth muscles of cranial arteries. The NSAIDs are effective in treating migraine of mild-to-moderate severity as migraine abortive drugs. However, it appears that they also slow down the progression of migraine from episodic to chronic.

Celecoxib has been available for many years as a capsule formulation, this selective inhibitor of cylcooxygenase-2 (COX-2). A study (90) compared celecoxib to naproxen 550 mg. The efficacy outcome measures were similar between the two treatments with fewer adverse events in the celecoxib group. Most recently, ELYXYB™ (a novel oral solution of celecoxib) was approved for the acute treatment of EM. Pharmacokinetic studies (91) compared 400 mg capsules of celecoxib and demonstrated that the maximum observed plasma concentrations (*C*_max_) of celecoxib after the administration of ELYXYB™ at three different doses were higher than for the 400-mg oral capsules. The *T*_max_ was within 1 h for ELYXYB™ compared to the capsules at 2.5 h. The area under the curve (AUCs) from 15 min to 2 h were three times higher for the liquid compared to the oral capsules. Trial data showed that a single dose of 120 mg of the oral solution provided 2-h relief in 35.6% of participants, compared to 21.7% with placebo. This oral solution provides yet another delivery option to patients limited to specific routes of administration. A placebo-controlled trial (92) found that 2-h post-dose pain freedom response rates were higher in the celecoxib oral solution group vs. placebo (32.8 vs. 23.5%; *P* = 0.020). The 2-h MBS freedom rates were also higher in the celecoxib oral solution group vs. placebo (58.1 vs. 43.9%; *P* = 0.003). A total of 10.7% of patients treated with celecoxib oral solution and 9.9% of placebo-treated patients reported a treatment-emergent adverse event (TEAE). Study drug-related TEAEs were reported by 7.3 and 7.4% of celecoxib oral solution and placebo patients, respectively.

## Conclusion

EM and CM constitute not only a debilitating disease for patients, but also a challenge for clinicians who treat them. Migraine sufferers are limited in daily activities due to bothersome associated symptoms like nausea. They can also be limited in tolerable treatment options. Additionally, patients with coexisting cardiovascular comorbidities have had limited options to treat their migraines. However, our arsenal of therapy has never been broader with the development of these new classes of drugs, along with new and improved deliveries of current therapies mixed with non-drug options. They may represent a new standard of care for migraine treatment. Conversations in a clinician's office may soon shift focus from struggling with control of symptoms to simply electing which therapy delivery type a patient prefers.

## Author contributions

BJ and FF contributed to performing the literature search and authorship of the paper. All authors contributed to the article and approved the submitted version.

## Conflict of Interest

FF has served as a consultant to Allergan/Abbvie, AgoneX Biopharmaceuticals Inc., Amgen, BioDelivery Sciences, Lundbeck, and Satsuma, has been on the Speakers Bureau for Allergan/Abbvie, Amgen, Lilly, and Teva, and has conducted research on behalf of Allergan/Abbvie, Amgen, Lundbeck, and Tx 360. The remaining author declares that the research was conducted in the absence of any commercial or financial relationships that could be construed as a potential conflict of interest.

## Publisher's Note

All claims expressed in this article are solely those of the authors and do not necessarily represent those of their affiliated organizations, or those of the publisher, the editors and the reviewers. Any product that may be evaluated in this article, or claim that may be made by its manufacturer, is not guaranteed or endorsed by the publisher.
